# Self-perception of weight status and its association with weight-related knowledge, attitudes, and behaviors among Chinese children in Guangzhou

**DOI:** 10.1016/j.je.2016.08.011

**Published:** 2017-02-20

**Authors:** Li Cai, Ting Zhang, Jun Ma, Lu Ma, Jin Jing, Yajun Chen

**Affiliations:** a Department of Maternal and Child Health, School of Public Health, Sun Yat-sen University, Guangzhou, China; b Institute of Child and Adolescent Health, School of Public Health, Peking University, Beijing, China

**Keywords:** Childhood obesity, Overweight, Weight perception, Knowledge-attitude-practice

## Abstract

**Background:**

How weight perception influences weight-related knowledge, attitudes, and behaviors in Chinese children is unknown. We investigated self-perception of body weight and its correlates, and analyzed the relationship between weight perception and weight-related knowledge, attitudes, and behaviors in children in Guangzhou, China.

**Methods:**

We assessed self-reported weight perception, weight-related knowledge, attitudes, and behaviors in 3752 children aged 7–12 years. Underweight or overweight was defined using the Chinese criteria based on body mass index (BMI). Binary logistic regression analyses were performed to assess correlates of weight underestimation.

**Results:**

In total, 27.3% of children underestimated and 6.7% overestimated their weight status. Weight underestimation was common among normal-weight (34.1%) and overweight children (25.3%). Older age, female sex, and child BMI z-score were negatively associated with normal-weight children's underestimation, whereas older age, paternal obesity, maternal obesity, and child BMI z-score were negatively associated with overweight children's underestimation. Correct answers on weight-related knowledge questions ranged from 81.5% to 98.6% and did not differ by weight perception within BMI categories. Although negative perceivers (i.e., those who perceived themselves as underweight or overweight) had a higher intention to change weight, they behaved more unhealthily on fruit intake, breakfast, screen time, and daily moderate-to-vigorous physical activities time than counterparts.

**Conclusion:**

Weight underestimation was prevalent in normal-weight and overweight children in Guangzhou. Negative perceivers had stronger willingness to change weight but tended to behave more unhealthily on certain behaviors than positive perceivers. Childhood obesity interventions should incorporate health education and practical support to promote healthy eating and physical activity.

## Highlights

• Over one third of children misperceived their weight status in Guangzhou, China.
• Underestimation of body weight was much more prevalent than overestimation.
• Underestimation was common in younger children, girls, and those with obese parents.
• Children perceived as underweight/overweight had a higher intention to change weight.
• Children perceived as normal weight behaved more healthily on certain behaviors.

## Introduction

The prevalence of childhood overweight and obesity has increased dramatically worldwide in recent years.^1^ With rapid economic growth over the past decades, China has also witnessed a sharp increase in childhood obesity.^2^ Childhood obesity may persist into adulthood,^3^ and it is associated with an increased risk of cardiovascular disease and type 2 diabetes mellitus.^4^ Therefore, childhood obesity prevention and intervention is of great concern to prevent future health consequences.

Although accurate self-perception of weight status is thought to be an important aspect of successful childhood obesity prevention and management, weight misperception has been repeatedly documented,^5^, ^6^, ^7^, ^8^, ^9^, ^10^ especially among overweight or obese individuals.^6^, ^8^ For example, a recent European study^8^ found that 42.9% of overweight/obese children underestimated their weight status. Studies from western countries reported that the accuracy of children's perception of weight status varied by socio-demographic and family factors, such as children's age,^8^ gender,^6^, ^8^ and parental weight status,^8^, ^11^ although the findings were not entirely consistent. Studies in the Chinese population also reported high prevalence of weight misperception.^12^, ^13^

Children's views about their weight status might influence their dietary intake and physical activity. Overweight or obese children who accurately perceived their weight status tended to report higher levels of weight loss behaviors, such as exercising to lose weight or dieting, than the misperceivers.^14^, ^15^ However, two studies focusing on overweight adolescents reported inconsistent results. One showed that the accurate perceivers had a combination of healthy and unhealthy weight control behaviors (i.e., dietary patterns and sedentary activities),^16^ and another one found that male accurate perceivers were less likely to report achieving recommended levels of fruit and vegetable intake and physical activity.^6^ Further research targeting children with different ages and different weight status is needed to determine the relationship between weight perception and changes of related behaviors.

China has the largest population of children in the world, and self-perception of weight status and its correlates in Chinese children may differ from those found in the West due to different socio-cultural backgrounds. A few studies have investigated the self-perception of weight status in Chinese younger-aged populations.^9^, ^12^, ^13^, ^17^, ^18^ However, none of these studies defined underweight or overweight using the Chinese criteria^19^, ^20^ consistent with East Asian ethnic characteristics. Additionally, most of these studies only focused on adolescents.^12^, ^13^, ^17^ Little is known about weight perception and its correlates in Chinese primary school children. To our knowledge, only one study has reported the association between weight perception and limited weight-related behaviors (e.g., eating sugar and snacks frequently and eating before sleep) in Chinese primary school children,^9^ and no study has assessed the relationship between weight perception and weight-related knowledge. The present study aims to investigate the self-perception of weight status, explore the socio-demographic correlates of underestimation, and examine the associations of weight perception with a range of weight-related knowledge, attitudes, and behaviors among Chinese children.

## Methods

### Study design and population

The cross-sectional data were retrieved from the baseline survey of a school-based obesity intervention program among Chinese children (“Development and Application of Student Critical Diseases Prevention and Control Technology and Related Standards”; Plan 1147).^21^, ^22^ A detailed description of the program was published elsewhere (Trial registration: January 22, 2015; Registration number: NCT02343588).^22^ The study was approved by the Ethics Committee of Peking University in China. Informed consent was obtained from all participants. This survey was conducted between September and November 2013.

A multistage random cluster sampling method was used to recruit the subjects in Guangzhou, a city in southern China. In the first stage, we selected four urban districts from the 10 districts of Guangzhou using the judgment sampling method. In the second stage, one or two primary schools were randomly selected from each of the four districts. Probability proportional to size sampling method was adopted in this stage, and a total of seven schools were chosen. In the third stage, all students in grades 2–5 were sampled from the selected schools. The principals were sent invitation letters and the research details. With the principals' permission, all students in grades 2–5 were invited to participate. Children were excluded if they were aged <7 years, or they were diagnosed with visceral diseases, abnormal growth and development, physical abnormality, or obesity caused by endocrine diseases or drugs. The response rates for schools and students were 100% (7/7 schools) and 78.4% (4942/6300 students), respectively. All individuals were asked to take a physical examination and answer a questionnaire. Participants who had no record of questionnaire (n = 690) or physical examination (n = 473), or had missing information about their age (n = 1), body mass index (BMI; n = 12), or self-reported weight perception (n = 14) were excluded, leaving 3752 children in the final analysis.

### Data collection

Student and parent questionnaires were administered to the whole class by well-trained investigators before the physical examination. One teacher supervised the survey procedure in each class. Students in grades 4–5 filled in the student questionnaire on their own in class, whereas students in grades 2–3 finished the student questionnaire under parents' guidance at home. Parent questionnaires were delivered by children to their parents and returned in 3 days. All questionnaires were checked for integrity and logicality upon return.

#### Socio-demographics

Demographic characteristics included children's date of birth, gender, number of siblings, parents' educational level, parents' occupations, and monthly household income. Parents self-reported their height (cm) and weight (kg). Potential correlates of children's weight underestimation were examined among these factors.

#### Self-perception of weight status

Children's self-perception of weight status was assessed by asking: “How do you feel about your current weight status?”, with five response options: “very thin”, “rather thin”, “average”, “rather fat”, and “very fat”. The responses were categorized into: perceived underweight (“very thin” and “rather thin”), normal weight (“average”), and overweight (“very fat” and “rather fat”). Children's self-perception was compared with actual weight status to assess underestimation, accurate estimation, and overestimation of body weight.

#### Actual weight status

Children's height (cm) and weight (kg) were measured by trained technicians following a standardized procedure. Height was measured using metal column height-measuring stands (200 cm long with 0.1 cm precision), and weight was measured using lever scales (weights to 120 kg with 0.1 kg precision). BMI (kg/m^2^) was calculated by dividing weight (kg) by height (m) squared. Underweight was defined based on the age- and gender-specific BMI cutoffs proclaimed by the National Health and Family Planning Commission of the People's Republic of China.^19^ Overweight was defined based on the age- and gender-specific BMI cutoffs recommended by the Working Group on Obesity in China in 2004.^20^ Parental BMI was calculated using self-reported height and weight. Their overweight was defined as BMI ≥ 24 kg/m^2^, whereas obesity was defined as BMI ≥ 28 kg/m^2^, in accordance with criteria for Chinese adults.^23^

#### Weight-related knowledge, attitudes, and behaviors

Weight-related knowledge was assessed with 10 items regarding dietary intake, exercise, and screen time. The responses of all items were: “true”, “false”, or “don't know”. Knowledge score was calculated by summing up the number of items which were correctly answered. Attitudes were evaluated with the questions: (1) “To what extent do you think obesity is bad for health?” (2) “Are you satisfied with your weight status?” (3) “Do you want to change your present weight status?” and (4) “Do you believe you can achieve an ideal weight status through effort?”. The responses were: “little”, “rather little”, “not sure”, “rather greatly”, or “greatly” for the first question and “no”, “rather no”, “not sure”, “rather yes”, or “yes” for the other three questions.

Weight-related behaviors included 10 items regarding dietary intake, physical activity, and screen time. Children reported frequency (days) and amount of fruit, vegetable, meat products, and sugar-sweetened beverages (SSBs) intake over the preceding 7 days. Average daily intakes of these foods were calculated as follows: average daily intake = [days × (amount in each of those days)]/7. Daily intake of fruit, vegetable, and meat products were then dichotomized according to dietary guidelines for Chinese children (2007).^24^ Children also reported frequency (days or times) of breakfast, high-energy snacks (e.g., chocolates and candies), fried food (e.g., fried chicken and fried potatoes), and western fast food (e.g., KFC and McDonald's) intake over the preceding 7 days. Additionally, children were asked about daily screen time (e.g., television viewing and computer using) and time of vigorous-intensity physical activities (e.g., running, basketball, football, and swimming) and moderate-intensity physical activities (e.g., cycling, table tennis, badminton, and calisthenics) over the preceding 7 days. Average daily time of moderate-to-vigorous-intensity physical activities (MVPA) was calculated by summing up daily time of the two kinds of physical activities. Daily screen time and MVPA time were dichotomized according to recommendations.^25^, ^26^

### Statistical analysis

Calculations were conducted using IBM SPSS software version 19.0 (IBM, Armonk, NY, USA). Continuous variables are presented as mean (standard deviation), whereas categorical variables are presented as number (percentage). For children's actual weight status, overweight and obesity were combined into a single category (overweight). Differences of continuous and categorical variables between different perceptions were evaluated using one-way ANOVA and Pearson Chi-Square tests, respectively. Binary logistic regression analyses were performed to assess the correlates of weight underestimation among normal-weight and overweight children. Potential correlates included child age, gender, and number of siblings; parents' educational level, occupations, monthly household income, and weight status; and child age- and gender-specific BMI z-score. In the regression analyses of normal-weight children, the overestimators were excluded because of small numbers (n = 164, 6.8% of normal-weight children). A two-sided *P* < 0.05 indicated statistical significance.

## Results

Table 1 summarizes the socio-demographic characteristics of the study population. In total, the mean age was 8.50 years old. Half of the children were boys (50.4%) and most were only children (76.3%). Over one-third of fathers and mothers had an education level of college or above (42.7% and 36.9%, respectively) and worked in commerce and service industries (37.6% and 37.0%, respectively). Approximately 23% of the participants had a monthly household income of <8000 RMB, whereas 36.7% refused to disclose household income.

**Table 1:** Socio-demographic characteristics of the population by children's weight perception.

Characteristics	Total (100%)	Self-perception of weight status (n = 3752)			*P*^a^
		Underweight (34.3%)	Normal weight (45.5%)	Overweight (20.1%)	
Age, years	8.50 (1.17)	8.37 (1.18)	8.48 (1.17)	8.77 (1.12)	0.000
Gender					0.000
Boys	1891 (50.4)	656 (50.9)	775 (45.4)	460 (60.8)	
Girls	1861 (49.6)	632 (49.1)	933 (54.6)	296 (39.2)	
Number of siblings					0.628
0	2784 (76.3)	955 (75.9)	1275 (76.7)	554 (76.0)	
1	737 (20.2)	260 (20.7)	323 (19.4)	154 (21.1)	
≥2	129 (3.5)	43 (3.4)	65 (3.9)	21 (2.9)	
Paternal educational level					0.299
Junior high school or below	370 (10.1)	124 (9.8)	159 (9.5)	87 (11.9)	
Senior high school	895 (24.4)	314 (24.9)	392 (23.5)	189 (25.7)	
Junior college	835 (22.8)	298 (23.7)	376 (22.5)	161 (21.9)	
College or above	1563 (42.7)	524 (41.6)	742 (44.5)	297 (40.5)	
Maternal educational level					0.068
Junior high school or below	459 (12.6)	156 (12.4)	188 (11.3)	115 (15.6)	
Senior high school	920 (25.2)	306 (24.4)	425 (25.5)	189 (25.7)	
Junior college	927 (25.4)	329 (26.6)	414 (24.9)	184 (25.0)	
College or above	1347 (36.9)	463 (36.9)	637 (38.3)	247 (33.6)	
Paternal occupation					0.312
Commerce and services	1355 (37.6)	437 (35.2)	636 (38.8)	282 (39.2)	
Professionals and technicians	750 (20.8)	264 (21.3)	350 (21.3)	136 (18.9)	
Administrators and clerks	740 (20.6)	263 (21.2)	328 (20.0)	149 (20.7)	
Other	755 (21.0)	277 (22.3)	326 (19.9)	152 (21.1)	
Maternal occupation					0.243
Commerce and services	1332 (37.0)	445 (35.9)	606 (37.0)	281 (38.7)	
Professionals and technicians	840 (23.3)	296 (23.9)	395 (24.1)	149 (20.5)	
Administrators and clerks	444 (12.3)	140 (11.3)	208 (12.7)	96 (13.2)	
Housewives	432 (12.0)	146 (11.8)	199 (12.2)	87 (12.0)	
Other	554 (15.4)	212 (17.1)	229 (14.0)	113 (15.6)	
Monthly household income					0.587^b^
<8000 RMB	839 (23.1)	280 (22.5)	383 (23.1)	176 (24.3)	
8000–14,999 RMB	769 (21.2)	261 (21.0)	346 (20.9)	162 (22.4)	
≥15,000 RMB	686 (18.9)	253 (20.4)	312 (18.8)	121 (16.7)	
Refuse to disclose	1331 (36.7)	449 (36.1)	617 (37.2)	265 (36.6)	

Continuous variables are presented as mean (standard deviation), whereas categorical variables are presented as number (percentage).

a Differences of continuous variables and categorical variables between different perceptions were evaluated using one-way ANOVA or Pearson Chi-Square tests, respectively.

b When those who refused to disclose were excluded from the analysis, the *P* value was 0.369.

Table 2 presents children's self-perception of weight status by actual weight status. In total, 66.0% of children accurately perceived their body weight. The percentages of accurate perception were 83.7% in underweight, 59.1% in normal-weight, and 74.7% in overweight children. Among normal-weight children, underestimation (34.1%) was much more prevalent than overestimation (6.8%). Normal-weight boys were more likely to underestimate themselves than girls (*P* < 0.001). Underestimation was also prevalent among overweight children (25.3%). eTable 1 shows children's self-perception of weight by actual weight status defined using the World Health Organization (WHO) criteria (2007).^27^ Weight underestimation was common among normal-weight (40.6%) and overweight children (30.2%).

**Table 2:** Self-perception of weight status by children's actual weight status.

Self-perception of weight status	Actual weight status			Total (100%)
	Underweight (14.5%)	Normal weight (64.4%)	Overweight (21.1%)	
Total (n = 3752)				
Underweight	458 (83.7)	824 (34.1)	6 (0.8)	1288 (34.3)
Normal weight	87 (15.9)	1427 (59.1)	194 (24.6)	1708 (45.5)
Overweight	2 (0.4)	164 (6.8)	590 (74.7)	756 (20.1)
Boys (n = 1891)				
Underweight	194 (84.3)	456 (40.4)	6 (1.1)	656 (34.7)
Normal weight	35 (15.2)	605 (53.5)	135 (25.4)	775 (41.0)
Overweight	1 (0.4)	69 (6.1)	390 (73.4)	460 (24.3)
Girls (n = 1861)				
Underweight	264 (83.3)	368 (28.6)	0 (0.0)	632 (34.0)
Normal weight	52 (16.4)	822 (64.0)	59 (22.8)	933 (50.1)
Overweight	1 (0.3)	95 (7.4)	200 (77.2)	296 (15.9)
*P*^a^	0.910	0.000	0.153	0.000

Values are presented as number (percentage).

a Differences of self-perception between boys and girls were evaluated using Pearson Chi-Square tests.

Table 3 displays the correlates of children's weight underestimation. Older age (adjusted OR 0.83; 95% CI, 0.75–0.91), female sex (adjusted OR 0.51; 95% CI, 0.41–0.63), and BMI z-score (adjusted OR 0.26; 95% CI, 0.22–0.30) were negatively associated with normal-weight children's underestimation, whereas older age (adjusted OR 0.59; 95% CI, 0.49–0.71), paternal obesity (adjusted OR 0.51; 95% CI, 0.27–0.96), maternal obesity (adjusted OR 0.17; 95% CI, 0.04–0.77), and BMI z-score (adjusted OR 0.30; 95% CI, 0.22–0.41) were negatively associated with overweight children's underestimation. After stratification into grades 2–3 and grades 4–5 groups, girls showed a negative association with weight underestimation among overweight children in grades 2–3 (adjusted OR 0.52; 95% CI, 0.29–0.94) but not in grades 4–5.

**Table 3:** Correlates of children's weight underestimation stratified by actual weight status.

	Normal-weight children (n = 2251)^a^		Overweight children (n = 790)	
	Crude OR	Adjusted OR^b^	Crude OR	Adjusted OR^b^
Age, years	0.91 (0.84–0.97)^∗∗^	0.83 (0.75–0.91)^∗∗^	0.66 (0.57–0.77)^∗∗^	0.59 (0.49–0.71)^∗∗^
Gender [reference: boys]				
Girls	0.59 (0.50–0.71)^∗∗^	0.51 (0.41–0.63)^∗∗^	0.82 (0.58–1.16)	0.79 (0.52–1.20)
Number of siblings [reference: 0]				
1	1.18 (0.95–1.46)	1.09 (0.83–1.44)	0.76 (0.49–1.17)	0.66 (0.38–1.14)
≥2	1.07 (0.68–1.69)	1.38 (0.75–2.54)	2.16 (1.00–4.66)	3.50 (1.19–10.29)^∗^
Paternal educational level [reference: college or above]				
Junior high school or below	1.25 (0.93–1.69)	1.12 (0.57–1.84)	0.66 (0.36–1.22)	0.60 (0.22–1.60)
Senior high school	1.14 (0.92–1.43)	1.09 (0.75–1.57)	0.85 (0.56–1.29)	0.85 (0.44–1.63)
Junior college	1.03 (0.82–1.29)	0.96 (0.70–1.32)	1.17 (0.78–1.76)	1.05 (0.60–1.84)
Maternal educational level [reference: college or above]				
Junior high school or below	1.17 (0.87–1.56)	0.98 (0.60–1.62)	0.69 (0.39–1.20)	0.68 (0.26–1.79)
Senior high school	1.12 (0.89–1.39)	1.11 (0.76–1.63)	1.08 (0.72–1.63)	1.28 (0.66–2.49)
Junior college	1.08 (0.86–1.35)	1.01 (0.74–1.37)	0.95 (0.62–1.44)	0.81 (0.47–1.41)
Monthly household income [reference: <8000 RMB]				
8000–14,999 RMB	1.07 (0.82–1.39)	1.20 (0.87–1.67)	0.93 (0.57–1.52)	0.71 (0.39–1.29)
≥15,000 RMB	1.01 (0.77–1.32)	1.04 (0.73–1.48)	0.88 (0.52–1.50)	0.70 (0.36–1.36)
Refuse to answer	0.97 (0.77–1.23)	1.01 (0.75–1.35)	1.01 (0.66–1.55)	0.87 (0.51–1.48)
Paternal weight status [reference: normal weight]				
Overweight	0.81 (0.67–0.97)^∗^	1.04 (0.83–1.31)	0.81 (0.57–1.15)	0.92 (0.60–1.40)
Obese	0.93 (0.66–1.31)	1.17 (0.76–1.79)	0.47 (0.28–0.80)^∗∗^	0.51 (0.27–0.96)^∗^
Maternal weight status [reference: normal weight]				
Overweight	0.78 (0.59–1.05)	1.09 (0.77–1.56)	0.67 (0.43–1.05)	0.95 (0.56–1.61)
Obese	0.72 (0.35–1.47)	1.29 (0.54–3.05)	0.16 (0.04–0.68)^∗^	0.17 (0.04–0.77)^∗^
Child BMI z-score	0.28 (0.24–0.31)^∗∗^	0.26 (0.22–0.30)^∗∗^	0.35 (0.27–0.45)^∗∗^	0.30 (0.22–0.41)^∗∗^

BMI, body mass index; OR, odds ratio.

Values are represented as odds ratios (95% confidence intervals) derived from binary logistic regression analyses.

^∗^*P* < 0.05, ^∗∗^*P* < 0.01.

a Among the normal-weight children, the under-estimators were compared with the accurate estimators. And those who overestimated their weight status were excluded from the analysis (n = 164, 6.8% of normal-weight children).

b All correlates were adjusted for each other and parental occupations.

Table 4 presents weight-related knowledge by weight perception in BMI categories. The correct rates of all items ranged from 81.5% for SSBs to 98.6% for fruits and vegetables. The correct rates of most knowledge items did not differ between different perceptions. However, for some items (e.g., SSBs and high-energy snacks in underweight children, fried food in overweight children, and exercise in normal-weight children), the accurate perceivers had a higher correct rate than the misperceivers (*P* < 0.05). In normal-weight children, overestimators had the highest knowledge score (*P* < 0.01).

**Table 4:** Children's weight-related knowledge by actual and perceived weight status.

Knowledge items	Underweight children (n = 547)		Normal-weight children (n = 2415)			Overweight children (n = 790)	
	Accurate (83.7%)	Overestimate^a^ (16.3%)	Underestimate (34.1%)	Accurate (59.1%)	Overestimate (6.8%)	Underestimate^b^ (25.3%)	Accurate (74.7%)
It is harmful to watch TV or play computer games for a long time. [yes]	419 (91.9)	76 (86.4)	734 (90.1)	1304 (91.7)	149 (91.4)	170 (86.7)	527 (90.1)
It is not necessary to exercise every day. [no]	404 (89.2)	76 (86.4)	712 (87.4)	1282 (90.7)	143 (87.7)^∗^	175 (88.4)	536 (91.5)
Fruits and vegetables should be eaten every day. [yes]	451 (99.3)	87 (97.8)	804 (98.4)	1397 (98.9)	161 (98.8)	196 (98.5)	570 (97.6)
Meat contains fat and protein and the more you eat meat, the better. [no]	369 (81.1)	71 (80.7)	685 (83.7)	1226 (86.4)	145 (89.0)	181 (91.0)	536 (91.6)
It is healthier to drink plain boiled water than sugar-sweetened beverages. [yes]	379 (83.5)	64 (71.9)^∗^	660 (80.4)	1174 (82.7)	136 (84.0)	158 (79.4)	470 (80.2)
You don't have to eat breakfast as long as you eat more lunch. [no]	448 (98.5)	85 (95.5)	811 (98.7)	1393 (97.8)	161 (98.8)	193 (97.0)	572 (97.3)
It is harmful to eat too much fried food. [yes]	413 (91.4)	78 (87.6)	753 (91.6)	1319 (92.8)	156 (95.7)	170 (86.3)	542 (92.2)^∗^
Western fast food (i.e, KFC, McDonald's, etc) is more nutritious. [no]	441 (96.9)	83 (93.3)	787 (95.9)	1378 (96.9)	159 (97.0)	192 (97.0)	564 (95.9)
It is beneficial to your health to drink milk every day. [yes]	433 (95.4)	82 (92.1)	795 (97.0)	1365 (96.0)	158 (96.3)	189 (95.5)	548 (93.7)
It is beneficial to your health to eat more high-energy snacks. [no]	447 (98.2)	84 (94.4)^∗^	794 (96.6)	1382 (97.1)	162 (98.8)	191 (96.0)	567 (96.4)
Knowledge score	10.0 (1.0)	9.0 (2.0)	10.0 (1.0)	10.0 (1.0)	10.0 (1.0)^∗∗^	10.0 (1.0)	10.0 (1.0)

Knowledge items are presented as number (percentage), whereas knowledge score is presented as median (interquartile range).

Differences of knowledge items between different perceptions within underweight, normal-weight, or overweight children were evaluated using Pearson Chi-Square tests, whereas differences of knowledge score between two or three perception groups were evaluated using Mann–Whitney U tests or Kruskal–Wallis tests, respectively.

^∗^*P* < 0.05, ^∗∗^*P* < 0.01.

a The overestimators in underweight group included 87 children who perceived themselves as normal weight and 2 children who perceived themselves as overweight.

b The underestimators in overweight group included 6 children who perceived themselves as underweight and 194 children who perceived themselves as normal weight.

Table 5 shows weight-related attitudes and behaviors by weight perception in BMI categories. Overall, nearly one-third of the participants were “not” or “rather not” satisfied with their weight status (32.9%), and approximately half had intention to change their present weight status (48.6%). In all three BMI categories, children who perceived themselves as normal weight were less likely to be unsatisfied with their weight status and had less desire to change it than their counterparts (*P* < 0.001).

**Table 5:** Children's weight-related attitudes and behaviors by actual and perceived weight status.

Questions/items	Underweight children (n = 547)		Normal-weight children (n = 2415)			Overweight children (n = 790)	
	Accurate (83.7%)	Overestimate^b^ (16.3%)	Underestimate (34.1%)	Accurate (59.1%)	Overestimate (6.8%)	Underestimate^c^ (25.3%)	Accurate (74.7%)
*Weight-related attitudes*							
To what extent do you think obesity is bad for health? [great or rather great]	330 (73.2)	58 (69.0)	625 (77.2)	1139 (80.7)	130 (70.7)	152 (77.6)	464 (79.6)
Are you satisfied with your weight status? [no or rather no]	270 (59.0)	14 (15.7)^∗∗^	352 (42.9)	115 (8.1)	90 (55.2)^∗∗^	28 (14.1)	364 (62.1)^∗∗^
Do you want to change your present weight status? [yes or rather yes]	310 (67.8)	26 (29.5)^∗∗^	446 (54.5)	346 (24.3)	125 (78.1)^∗∗^	69 (35.6)	490 (83.9)^∗∗^
Do you believe you can achieve an ideal weight status through effort? [yes or rather yes]	285 (62.5)	62 (69.7)	507 (61.7)	1008 (71.5)	118 (72.8)^∗∗^	148 (75.1)	408 (69.5)
*Weight-related behaviors [reaching rate]*							
Fruit [daily intake ≥2 servings^a^/day]	91 (20.6)	28 (32.6)^∗^	183 (23.6)	374 (27.2)	43 (27.9)	51 (26.3)	150 (26.6)
Vegetable [daily intake ≥3 servings^a^/day]	74 (16.4)	20 (22.7)	141 (17.3)	296 (21.1)	34 (21.0)	31 (16.0)	137 (23.6)^∗^
Meat products [daily intake ≤1 serving^a^/day]	285 (62.4)	58 (66.7)	528 (64.3)	912 (64.1)	96 (58.9)	130 (65.3)	349 (59.7)
Breakfast [frequency = 7 day/week]	431 (94.3)	82 (93.2)	769 (93.6)	1348 (94.7)	145 (89.0)^∗^	188 (94.0)	551 (93.5)
SSBs [daily intake = 0 cups/week]	227 (50.3)	46 (53.5)	361 (44.5)	619 (44.0)	72 (44.7)	77 (39.1)	220 (37.7)
High-energy snacks [frequency = 0 day/week]	135 (29.5)	33 (37.5)	285 (34.7)	451 (31.8)	60 (36.8)	72 (36.0)	198 (33.9)
Fried food [frequency = 0 times/week]	313 (68.9)	55 (62.5)	522 (63.6)	885 (62.5)	106 (65.0)	124 (62.0)	337 (57.4)
Western-style fast food [frequency = 0 times/week]	213 (47.0)	45 (52.3)	359 (44.0)	629 (44.4)	79 (48.8)	86 (43.0)	233 (39.9)
Screen time [daily time ≤ 2 h/day]	387 (87.8)	74 (86.0)	671 (84.0)	1180 (85.8)	124 (77.0)^∗^	160 (81.6)	458 (79.5)
MVPA [daily time ≥ 1 h/day]	113 (27.7)	31 (38.3)	243 (33.8)	486 (37.6)	48 (32.0)	83 (46.6)	176 (33.7)^∗^

SSBs, sugar-sweetened beverages; MVPA, moderate to vigorous physical activity.

All values are presented as number (percentage).

Differences of items between different perceptions within underweight, normal-weight, or overweight children were evaluated using Pearson Chi-Square tests.

^∗^*P* < 0.05, ^∗∗^*P* < 0.01.

a A serving of fruit or vegetable was equivalent to 100 g, whereas a serving of meat products was equivalent to 75 g.

b The overestimators in underweight group included 87 children who perceived themselves as normal weight and 2 children who perceived themselves as overweight.

c The underestimators in overweight group included 6 children who perceived themselves as underweight and 194 children who perceived themselves as normal weight.

Only 25.6% and 19.9% of participants met the recommendation for fruit (≥2 servings/day) and vegetable consumption (≥3 servings/day), respectively. Less than half did not consume SSBs (43.9%) and high-energy snacks (33.1%). A majority of children met the recommendation for screen time (84.0%), but only one-third reached the recommendation for MVPA (35.2%). We found no statistical difference of the reaching rates of most behaviors between different perceptions. However, underweight children with accurate perception had a lower reaching rate of fruit intake and a critical lower reaching rate of MVPA than the overestimators (*P* < 0.05 and *P* = 0.056, respectively). Normal-weight children who overestimated themselves had a lower reaching rate of breakfast and screen time than their counterparts (*P* < 0.05). In overweight children, the accurate perceivers had a higher reaching rate of vegetable intake but a lower reaching rate of MVPA than the underestimators (*P* < 0.05).

## Discussion

This study demonstrated that over one-third of children misperceived their weight status in Guangzhou, China. Underestimation of body weight was much more prevalent than overestimation. Underestimation was more common among younger children, boys, and those with a lower BMI z-score, but was less likely to occur when parents were obese. Perceiving oneself as overweight or underweight was associated with stronger weight dissatisfaction and a higher intention to change weight. However, children who perceived themselves as overweight or underweight, regardless of actual weight status, tended to behave more unhealthily on certain weight-related behaviors.

A large proportion of children (34.0%) misperceived their body weight, especially among normal-weight individuals. Nearly 41% of normal-weight children misperceived themselves as thin or fat, while about 25% of overweight children considered their weight as average or thin. Results were similar to observations reported by Zhao et al,^9^ which showed that misperception was more prevalent in normal-weight children in China. In western countries,^5^, ^11^ however, normal-weight children had a higher accuracy of weight perception than overweight children. This discrepancy might be explained by diverse social norms of ideal weight in different ethnic and cultural backgrounds. Furthermore, over a third of normal-weight children underestimated their weight status, which was much higher than the corresponding rate in American healthy-weight children (about 10%).^5^ Weight underestimation may result in lack of motivation to avoid weight gain^28^ and was associated with excessive consumption of certain high-energy food (e.g., sugar and snacks) and bad eating habits (e.g., monophagia and eating before sleep), thus increasing the likelihood of weight gain.^9^ Meanwhile, a quarter of overweight children underestimated their body weight, which was similar to the findings of another study in Chinese children^9^ but lower than rates in American (74% in overweight and 42% in obese^5^) and European children (43%).^8^ Taken together, our findings showed that weight underestimation occurred more often when a child was normal weight rather than overweight, indicating the importance of promoting accurate awareness of weight status both in normal weight and overweight Chinese children.

Factors associated with weight misperception are not well-understood among Chinese children. In the present study, child age, gender, BMI z-score, and parental obesity were identified as important correlates of weight underestimation. Younger children were more likely to underestimate their body weight, which was in line with some^8^, ^29^ but not all previous studies.^18^, ^30^ Older children may have been exposed to more mass media messages and education,^14^ thus gaining a better understanding of obesity. In addition, normal-weight girls were less likely to underestimate themselves than normal-weight boys, which was in accordance with other studies.^6^, ^8^, ^16^, ^31^, ^32^, ^33^ This may be due to girls being more susceptible to the influence of media on physical appearance, which advocates slimness for girls and may affect their own weight standards. Another interesting finding was that parental obesity was negatively associated with overweight children's underestimation, which was contrary to the findings of previous studies from other countries.^8^, ^11^ Maximova et al^11^ reported that children living with overweight/obese parents were more likely to underestimate their body weight. Our findings suggested that children of obese parents might more accurately perceive their own body image than children of non-obese parents.

Previous studies have linked weight perception to weight control behaviors in children,^14^, ^15^ so we analyzed the associations of weight perception with weight-related attitudes and behaviors in all BMI categories. In general, the subjects had a good awareness of most knowledge items, except for the harmfulness of SSBs. Considering that SSBs intake increases the risk of obesity^34^ and over half of children consumed SSBs in this study, we recommend that health education about SSBs should be enhanced among primary school students. To our knowledge, few studies have examined weight perception in relation to weight-related knowledge in Chinese children. Surprisingly, we found no difference in the correct rates of most items between different perceptions. One possible reason was that primary school students received health education through similar channels, such as school curricula, books, and media. It was noteworthy that, among the items which varied significantly across self-perceptions, children with accurate weight perception were better aware of the knowledge items. This was plausible, since children who hold accurate views on their weight status may also have better awareness of weight-related knowledge.

In agreement with other studies,^9^, ^12^ we observed a significantly greater weight dissatisfaction and a stronger desire to change present weight status among overweight and underweight children with accurate perception. These results suggest that weight dissatisfaction is strongly correlated with perceived weight status. Concordant weight perception in overweight children might help raise weight dissatisfaction and improve the intention to maintain healthy weight. Nonetheless, the overweight and underweight children who accurately perceived themselves were less likely than misperceivers to believe they could achieve an ideal weight status through their own effort. Those who have no confidence in weight change may lack persistent motivation for subsequent weight control behaviors, so encouragement is required to promote children's engagement in weight change.

Like previous studies,^9^, ^16^, ^17^, ^35^ we observed relationships between weight perception and weight-related behaviors. Specifically, overweight children with accurate perception were less likely to meet MVPA recommendations^26^ than those who underestimated themselves, whereas normal-weight children with accurate perception were more likely to eat breakfast every day and meet screen time recommendations^25^ than their counterparts who overestimated themselves. A hint from this study was that negative perceivers, whether accurate or not, tended to behave more unhealthily than positive perceivers on those weight-related behaviors, such as long screen time and short time of MVPA. Similar results were shown in other studies. Xie et al found that girls who perceived or misperceived themselves as overweight were more likely to consume more snack foods and less milk and dairy products, and girls who perceived or misperceived themselves as underweight ate fewer vegetables and fruits and were less likely to engage in vigorous physical activities.^17^ Another Chinese study reported that normal-weight children with misperceived fatness or thinness were more likely to adopt unhealthy weight-related behaviors, such as eating before bed and eating sugar and snacks frequently, compared with those with accurate weight perceptions.^9^ Furthermore, a study conducted among Australia adolescents showed that overweight and obese accurate perceivers were more likely to be physically active but did not show healthier dietary patterns or less time in sedentary activities.^16^ An American study found that adolescents who tried to lose weight did not have higher fruit and vegetable consumption or higher physical activity compared with the others.^35^ Wang et al suspected that those who tried to lose weight did not actually make the desirable behavioral changes; they initiated the changes but failed to maintain them, or the changes were too small to achieve more desirable eating and physical activity patterns compared with their counterparts.^35^ Psychosocial health problems (e.g., feeling stressful, depression, and poorer appetite) induced by perceived thinness or fatness^13^ might also be considered as a barrier to the adoption of healthy weight-related behaviors. Therefore, in addition to general education about weight perception, a healthy diet- and physical activity-friendly environment is particularly needed to promote desirable behavior in children. Moreover, longitudinal studies are warranted to examine the influence of weight perception on weight-related behaviors over time.

This study has some limitations, due to which the findings must be interpreted carefully. First, the cross-sectional design precluded the identification of casual associations. Second, a substantial proportion of children did not participate in the study or were excluded from the final analysis, which might result in selection bias in this study. However, no difference was found in the distribution of age or gender between the participants and non-participants, indicating that selection bias stemming from non-participation might be small. Third, the children in grades 2–3 completed the student questionnaire under parents' guidance, so their response might be influenced by parents. Furthermore, we did not take into account other possible correlates of weight perception, such as exposure to social media,^17^ social comparison, and psychological factors.^36^ Finally, the self-reported weight-related behaviors were not objectively measured and might be subject to recall bias.

## Conclusions

This study showed that underestimation of weight status was common among normal-weight and overweight children in Guangzhou, China. Children who negatively perceived themselves had stronger weight dissatisfaction and willingness to change their body weight. However, they tended to behave more unhealthily on certain weight-related behaviors than positive perceivers. Intervention for childhood obesity should incorporate education about accurate identification of actual weight status and practical strategies for promoting healthy eating and physical activity.

## Conflicts of interest

None declared.

## Appendix A Supplementary data

eTable 1. Self-perception of weight status by children’s actual weight status defined using the World Health Organization criteria.
