# Supplementary material for: Self-perception of weight status and its association with weight-related knowledge, attitudes, and behaviors among Chinese children in Guangzhou
Source: J Epidemiol. 2017 Feb 20;27(7):338–45. doi: 10.1016/j.je.2016.08.011 (PMC5498423; doi:10.1016/j.je.2016.08.011)
Supplement: Supplementary file 1 — eTable 1. Self-perception of weight status by children’s actual weight status defined using the World Health Organization criteria. [file mmc1.pdf]

eTable 1. Self-perception of weight status by children's actual weight status defined using the World Health Organization criteria[a]

Self-perception of weight status	Actual weight status			Total
	Underweight (6.3%)	Normal weight (69.7%)	Overweight (24.0%)	(100%)
Total (n=3,752)				
Underweight	218 (92.4)	1,061 (40.6)	9 (1.0)	1,288 (34.3)
Normal weight	18 (7.6)	1,427 (54.6)	263 (29.2)	1,708 (45.5)
Overweight	0 (0)	126 (4.8)	630 (69.8)	756 (20.1)
Boys (n=1,891)				
Underweight	94 (94.0)	553 (47.2)	9 (1.5)	656 (34.7)
Normal weight	6 (6.0)	575 (49.1)	194 (31.3)	775 (41.0)
Overweight	0 (0)	43 (3.7)	417 (67.3)	460 (24.3)
Girls (n=1,861)				
Underweight	124 (91.2)	508 (35.2)	0 (0.0)	632 (34.0)
Normal weight	12 (8.8)	852 (59.0)	69 (24.5)	933 (50.1)
Overweight	0 (0)	83 (5.8)	213 (75.5)	296 (15.9)
P [b]	0.419	0.000	0.011	0.000

Values are presented as number (percentage).

[a] World Health Organization. Growth reference 5-19 years. Geneva: World Health Organization; 2007. Available from: http://www.who.int/growthref/who2007_bmi_for_age/en/. Accessed 8 Oct, 2015.

[b] Differences of self-perception between boys and girls were evaluated using Pearson Chi-Square tests
